# Changes in fibroblast growth factor 21 levels associated with alcohol consumption and smoking cessation

**DOI:** 10.1530/EC-25-0713

**Published:** 2026-01-05

**Authors:** Clara O Sailer, Cyril F Vogel, Sophie Monnerat, Leila Probst, Mirjam Christ-Crain, Bettina Winzeler, Julie Refardt

**Affiliations:** ^1^ Department of Endocrinology, Diabetology and Metabolism, University Hospital Basel, Basel, Switzerland; ^2^ Division of Endocrinology, Diabetes and Hypertension, Brigham and Women’s Hospital, Harvard Medical School, Boston, Massachusetts, USA; ^3^ Department of Endocrinology, Diabetology and Clinical Nutrition, University Hospital Zurich, Zurich, Switzerland; ^4^ Department Internal Medicine, Section of Endocrinology, Erasmus Medical Center, Rotterdam, The Netherlands

**Keywords:** FGF21, glucagon-like peptide 1 receptor agonist, metabolic changes, liver disease

## Abstract

**Background:**

Alcohol consumption was shown to increase endogenous fibroblast growth factor 21 (FGF21), but knowledge about the effect of alcohol cessation on FGF21 is limited. The effects of cigarette smoking and cessation on FGF21 levels are unknown. The objective of this study was to investigate moderate alcohol and cigarette consumption and their cessation on FGF21 levels.

**Methods:**

This is a secondary analysis of two prospective intervention studies. Study 1: ten healthy men undergoing a beer or water intervention with blood sampling over 720 min. Differences in FGF21 levels between alcohol and water intake were assessed using a mixed-effect model. Study 2: 144 alcohol-drinking men or women undergoing a 12-week intervention of glucagon-like peptide 1 (GLP-1) receptor agonist dulaglutide vs placebo on smoking and alcohol cessation. Differences in FGF21 levels after 12 weeks of treatment with GLP-1 in persistent drinkers/smokers compared to those who had stopped drinking/smoking, were assessed using mixed-effect models.

**Results:**

Study 1: FGF21 levels at 240 min following beer intake were higher compared to water intake (1.386.0 pg/mL (95% CI: 934.55; 1,837.44), *P* < 0.001). Study 2: participants who stopped drinking alcohol had lower FGF21 levels compared to persistent drinkers (−228.65 pg/mL (95% CI: −440.4; −14.6), *P* = 0.03). Smoking cessation had no effect on FGF21 levels (*P* = 0.13).

**Conclusion:**

Our findings demonstrate a dynamic response in FGF21 levels, with acute moderate alcohol consumption inducing elevated FGF21 levels, and cessation of drinking lowering FGF21 levels, indicative of potential liver recovery. No effect of cigarette smoking cessation on plasma FGF21 levels was observed.

## Introduction

Alcohol consumption is a major health burden and is ranked one of the leading risk factors for loss in disability-adjusted life years (DALYs) (1) by inducing liver diseases, pancreatitis, cardiometabolic complications, pneumonia, and malignancy (2). How alcohol influences metabolism is actively being investigated, and improved understanding of the underlying biology may prove helpful in identifying pharmacological targets and treatment strategies for alcohol cessation (3).

Fibroblast growth factor 21 (FGF21) is a hormone primarily released into the bloodstream by the liver upon metabolic stress and dietary imbalances, e.g., starvation, increased intake of glucose, fructose, and ethanol, with the latter being the most potent inducer of FGF21 (4). The effects attributed to FGF21 have rapidly expanded, including reducing oxidative stress and lipogenesis, increasing hepatic insulin sensitivity, and promoting fatty acid β-oxygenation, thus preventing liver damage (5). In humans, pathological conditions such as obesity, insulin resistance, type 2 diabetes mellitus (T2DM), and liver injury are associated with increased levels of FGF21 (4). Data show increasing levels of FGF21 upon acute and chronic alcohol intake, as well as a correlation of FGF21 with the amount of alcohol consumed (6). FGF21 knockout mice demonstrate significantly increased alcohol consumption compared to wild-type mice (7, 8, 9, 10, 11, 12), while mice with transgenic overexpression of FGF21 show reduced alcohol consumption (8).

Furthermore, cigarette smoking is one of the most common addictions, leading to respiratory diseases, cardiovascular diseases, and malignancy. Cigarette smoking represents one of the most prevalent causes of preventable morbidity and mortality (13). The association between FGF21 and cigarette smoking is poorly understood, but emerging evidence suggests an association between smoking and elevated FGF21 levels in humans (14, 15). There is limited evidence of the effect of smoking cessation on FGF21 levels, but current studies suggest that it has no effect (16).

Glucagon-like peptide-1 receptor agonists (GLP-1 RAs) are established therapies for type 2 diabetes and obesity, exerting beneficial effects on glycemic control and body weight. Recent preclinical and translational studies have identified FGF21 as a key mediator of GLP-1 RA-induced metabolic improvements, including attenuation of hepatic glucose output and regulation of lipid metabolism (17, 18, 19). While GLP-1 RAs such as liraglutide and exenatide have been shown to stimulate hepatic FGF21 expression and increase circulating FGF21 in animal models, the mechanisms and clinical relevance of this axis remain incompletely defined (20, 21).

In summary, changes in FGF21 level upon acute and chronic alcohol consumption, smoking cessation, as well as treatment with GLP-1 receptor agonists, are limited. Accordingly, we aimed to investigate the effects of acute and chronic alcohol consumption, smoking cessation, and treatment with the GLP-1 RA dulaglutide on plasma FGF21 levels to improve the understanding of FGF21 biology.

## Materials and methods

### Participants and study design

This is a secondary analysis of two prospective interventional studies conducted at a tertiary care center.

**Study 1:** prospective, pathophysiological, single-center, crossover study investigating healthy men during moderate, acute alcohol consumption between February and April 2019. Eligible participants were between 20 and 40 years old and had a normal body mass index (BMI 18–25 kg/m^2^). Exclusion criteria included any acute or chronic illness, a history of alcohol use disorder, alcohol intake of >2 standard drinks per day (1 standard drink was defined as either 33 cL of 5% beer, 13 cL of wine, or 4 cL of 40% liquor (22)), active nicotine use, participation in another clinical study with investigational drugs within the past 30 days and during the study. For further details, please review the original publication.

**Study 2:** prospective, double-blind, randomized, placebo-controlled, single-center study investigating the effect of the glucagon-like peptide-1 receptor agonist (GLP1-RA) dulaglutide compared to placebo in addition to standard of care (varenicline and behavioral counseling) on cigarette smoking cessation after 12 weeks. The study was conducted between June 2017 and June 2022. Eligible participants were between 18 and 75 years old, were daily smokers with at least moderate cigarette dependency (Fagerstroem score (craving for cigarette smoking score) of ≥5 points), and were willing to participate in a cigarette smoking cessation program. For this sub-analysis, participants had to consume at least one standard drink containing alcohol per week at baseline and have a full set (basal and 12-week follow-up visit) of frozen plasma samples available for the analysis of FGF21 levels. Exclusion criteria were most notable for pregnancy, pre-existing treatment with any GLP1-RA, history of pancreatitis, severe renal insufficiency (estimated glomerular filtration rate <30 mL/min/1.73 m^2^), unstable psychiatric conditions, and anorexia nervosa. For further details, please consult the original publication.

All participants provided written informed consent before the studies after receiving a comprehensive explanation of the purpose and nature of all procedures used during the studies. Both study protocols were approved by the Ethical Committee of Northwest and Central Switzerland and registered on ClinicalTrials.gov and Study 2 was approved by the national agency for the authorization and supervision of therapeutic products (Swissmedic, Switzerland). The studies were performed according to the guidelines of good clinical practice.

### Study procedures

**Study 1:** participants arrived at 18:00 h at the study center with no food or liquid consumed three hours prior, alcohol and nicotine were prohibited 24 h before the study, and sport was prohibited on test days. An indwelling venous catheter was placed for repeated blood draws, and a glass of water (250 mL) with a standardized meal (sandwich) was provided. The baseline blood samples were drawn at 19:00 h and followed by one of the following two interventions: alcohol-intervention, a weight-based amount of beer containing 5% alcohol (0.8 g alcohol per kg body weight), calculated using the Widmark formula, was given to reach a blood alcohol concentration (BAC) of 0.8%; water-intervention, the same amount of water as given during the alcohol-intervention was provided, ensuring that fluid was consumed equally on both days.

To ensure continuous fluid intake, one-third of the total volume had to be consumed every 20 min. Blood samples and vital signs were collected at baseline, 60, 90, 150, 240, and 720 min. Participants consumed no food or fluids beyond what was specified in the test protocol. Test days were completed in random order, with at least 48 h between the test days.

**Study 2:** baseline data, including information on smoking and alcohol consumption (number of standard drinks per week), were collected at the start of the study, followed by a blood draw. Afterward, participants started treatment with varenicline, behavioral counseling by a smoking cessation service, and were randomized to either dulaglutide 0.75 mg for 1 week followed by 1.5 mg weekly or placebo for a total of 12 weeks. Visits at the study center with weekly injections of the study drug. At week 12, self-reported smoking status, self-reported alcohol consumption, blood and urinary analyses including cotinine measurement were documented (smoking abstinence at week 12 was defined as self-reported 7-day smoking abstinence and end-expiratory exhale carbon monoxide measurements of 10 ppm or less). Data from baseline and week 12 were included in this sub-analysis.

### Laboratory measurements

**Study 1:** plasma sodium, serum BAC, and plasma FGF21 were measured at each time point. Plasma sodium and serum BAC measurements were performed immediately in nonfrozen samples by automated biochemical analyses at the central laboratory of the University Hospital Basel, Switzerland.

**Study 2:** blood samples were collected at baseline and at the 12-week visit, centrifuged and stored at −70°C for further analyses.

For both studies, FGF21 was assessed in a batch analysis from frozen plasma. Most samples were thawed for the first time. FGF21 was measured using the U-PLEX Human FGF-21 Assay, an electro-chemiluminescent sandwich immunoassay (K1515WK-2, Meso Scale Diagnostics, USA). The lower limit of detection is 2.8 pg/mL (range 2.0–3.0), and intra-run coefficient of variation (CV) was 4.3% and inter-run CV 12.1%.

### Study objectives and outcomes

The overall objective of this post hoc secondary analysis was to improve the physiological understanding of alcohol consumption and cigarette smoking on plasma FGF21 levels. The primary objective was to investigate the effect of acute alcohol consumption on plasma FGF21 levels. Secondary objectives were the effect of alcohol consumption and smoking cessation on plasma FGF21 levels, as well as the association of FGF21 with the GLP-1 RA dulaglutide.

**Study 1:** the primary endpoint was the mean difference in plasma FGF21 levels over all time points after moderate alcohol compared to water intervention.

**Study 2:** the primary endpoint was the mean difference in plasma FGF21 levels from baseline to week 12 between participants who stopped drinking alcohol compared to those who continued drinking alcohol. Secondary endpoints were the mean difference in plasma FGF21 levels from baseline to week 12 between participants who stopped smoking cigarettes compared to those who continued cigarette smoking, and between those who received the GLP-1 RA dulaglutide compared to placebo.

### Statistical analyses

For descriptive statistics, discrete variables are presented as frequencies (*n*) with percentages (%), while continuous variables are presented as either mean with standard deviation (SD) or median with interquartile range (IQR), depending on the data distribution.

**Study 1:** to estimate the difference in plasma FGF21 levels between the alcohol and water intervention over time, a linear mixed-effect model was used with FGF21 levels as the outcome variable and intervention (alcohol vs water) and time point with and without their interaction as fixed effects, and participants as a random effect. An analysis of variance (ANOVA) comparing the different models, along with their respective Akaike information criterion (AIC), was used to identify the model that best explained the data. As a secondary analysis, glucose was modeled as a fixed effect to control for its potential confounding influence on FGF21 levels.

**Study 2:** to estimate the difference in plasma FGF21 levels from baseline to week 12 between the participants who (a) stopped drinking alcohol and (b) stopped smoking cigarettes compared to those who (a) continued drinking alcohol and (b) continued smoking cigarettes, we used a linear mixed-effect model with FGF21 levels as the outcome variable and (a) alcohol or (b) cigarette smoking as a binary (yes/no) variable as fixed effects, and participants as random effects. To estimate the difference in plasma FGF21 between the GLP-1 RA dulaglutide compared to placebo, we used a linear mixed-effect model with FGF21 levels as the outcome variable and intervention (dulaglutide vs placebo) as a fixed effect, and participants as random effects. To evaluate confounding variables, age, sex, and BMI were added as fixed effects.

A two-sided *P* value of <0.05 was considered statistically significant. Analyses were performed using R statistical software (version 4.3.3), and figures and tables were created using R statistical software (version 4.3.3).

## Results

### Acute effect of moderate alcohol consumption on plasma FGF21 levels

**Figure 1:**
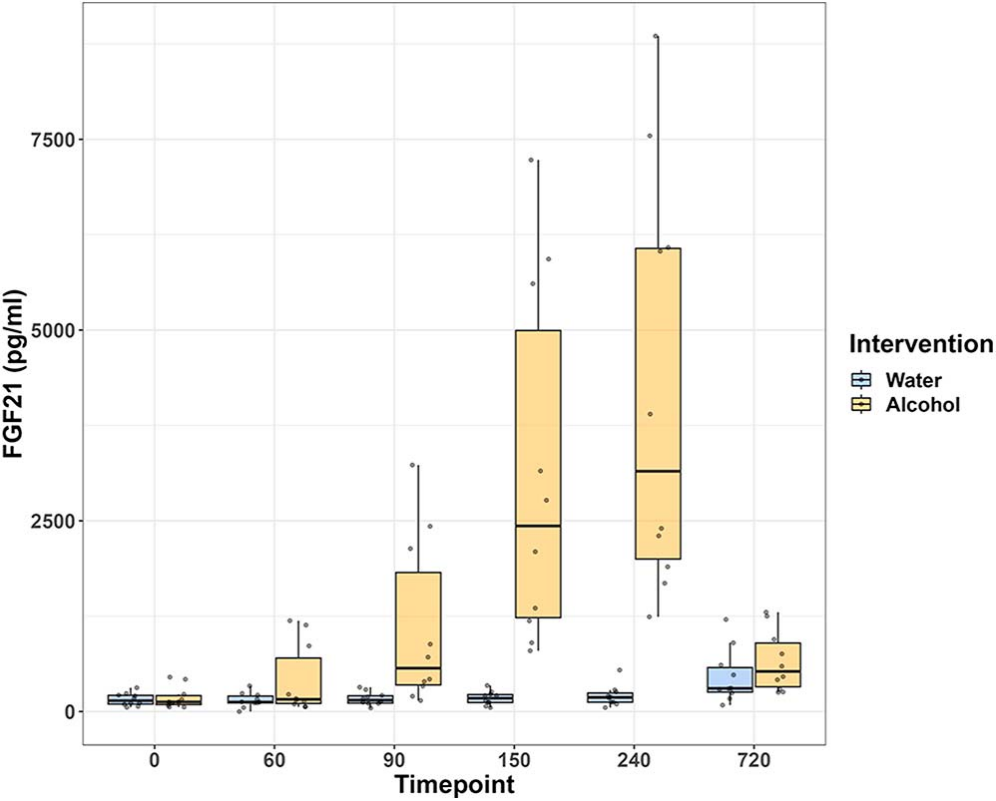
Study 1: FGF21 levels by intervention over time. Boxplots represent median (horizontal line) and IQR (25th and 75th percentile, outer box). Whiskers extend to 1.5 times the interquartile range. Single dots represent individual data points. A linear mixed-effect model was calculated with FGF21 levels as the outcome variable, and intervention (alcohol vs water) and time point as fixed effects, as well as their interaction, and participants as a random effect.

**Study 1:** ten healthy men with a median age of 24 years (IQR 22; 31) and a median alcohol consumption of five standard drinks per week (IQR 4; 7) were included. At baseline, plasma sodium and osmolality levels were within the normal range. Median beer consumed to reach the aimed BAC of 0.8% was 1,500 mL (IQR 1,500; 1,600). BAC peaked 90 min after the start of alcohol consumption with a median of 0.85% (IQR 0.7, 0.9) and returned to 0.0% after 720 min in all participants. No increase in BAC was observed after water consumption. Following alcohol consumption, plasma FGF21 levels increased from 126 pg/mL (IQR 77; 275) at baseline to a maximum of 3,150 pg/mL (IQR 1,844; 6,449) at 240 min and decreased to 525 pg/mL (IQR 284; 1,022) at the end of the observation period. After consuming an equal amount of water, plasma FGF21 levels increased from 142 pg/mL (IQR 878; 220) to 185 pg/mL (IQR 116; 263) at 240 min and rose further to 303 pg/mL (IQR 225; 682) at the end of the observation period. Changes in plasma FGF21 levels over all time points are shown in Table 1 and Fig. 1. In a mixed-effect model, FGF21 levels were significantly higher following alcohol consumption with an estimated difference of 1,386 (95% CI: −1,837, −935) pg/mL, *P* < 0.001, AIC 2,101, and notable increases at time points 150 min (estimated difference 1,469, 95% CI: 705, 2,232; *P* < 0.001) and 240 min (estimated difference 2,029, 95% CI: 1,266, 2,793; *P* < 0.001), AIC 2,074. There was a statistically significant interaction between time point and intervention (*P* < 0.001), AIC 2,023. The model using interaction best explained the data (*P* < 0.001). Adjusting the model for glucose did not change the results, and glucose was not an independent predictor of FGF21 levels (glucose estimated difference 91, 95% CI: –200, 382; *P* = 0.56).

**Table 1:** Study 1: FGF21 in response to acute alcohol intake.

	Water-intervention	Alcohol-intervention
	*n* = 10	*n* = 10
**Plasma FGF21, pg/mL**		
Baseline	142.0 (87.7; 219.9)	125.5 (77.4; 275.2)
60 min	125.5 (113.1; 226.4)	159.7 (89.3; 928.5)
90 min	149.1 (106.5; 230.5)	568.7 (300.4; 2,208.7)
150 min	175.1 (105.4; 234.4)	2,432.3 (1,117.1; 5,688.4)
240 min	184.8 (116.3; 263.4)	3,149.6 (1,843.8; 6,448.7)
720 min	302.7 (225.0; 681.9)	525.3 (284.3; 1,021.7)
**Blood alcohol concentration, %**		
Baseline	0.0 (0.0, 0.0)	0.0 (0.0, 0.0)
60 min	0.0 (0.0, 0.0)	0.6 (0.5, 0.7)
90 min	0.0 (0.0, 0.0)	0.9 (0.7, 0.9)
150 min	0.0 (0.0, 0.0)	0.9 (0.7, 0.9)
240 min	0.0 (0.0, 0.0)	0.6 (0.5, 0.7)
720 min	0.0 (0.0, 0.0)	0.0 (0.0, 0.0)

Data are presented as medians and interquartile ranges.

### Effect of alcohol consumption and smoking cessation on FGF21 levels

**Study 2:** out of 255 participants enrolled in the initial study, 151 drank alcohol at baseline, of whom 149 had a complete set of frozen plasma (baseline and 12 weeks). Five participants were excluded due to measurement errors or outliers, leaving a total of 144 subjects for the present analysis. Baseline characteristics of this subset are shown in Table 2. Fifty-five (38.2%) participants were male, and mean age was 43 years (SD 13). Median alcohol consumption was three standard drinks per week (IQR 2; 7). Median amount of total cigarette smoking was 20 pack-years (IQR 11; 34) and median current cigarette smoking was 20 cigarettes per day (IQR 14; 20). Seventy-one participants (49.3%) were randomized to dulaglutide and 73 participants (51.7%) to placebo. Baseline characteristics did not differ significantly between the two groups (see Table 2).

**Table 2:** Study 2: baseline characteristics.

	Participants *n* = 144
Age, years (mean (SD))	42.8 (13.2)
Sex, male (%)	55 (38.2)
Ethnicity (%)	
Caucasian	142 (98.6)
Amount of smoking, cigarettes/day (median (IQR))	20 (14, 20)
Pack years (mean (SD))	20 (11, 34)
Amount of alcohol, glasses/week (median (IQR))	3 (2, 7)
Systolic BP, mmHg (mean (SD))	120 (14)
Diastolic BP, mmHg (mean (SD))	78 (8)
Heart rate, bpm (mean (SD))	74 (10)
BMI, kg/m^2^ (mean (SD))	26.7 (5.0)
HbA1c, % (mean (SD))	5.4 (0.5)
FGF 21, pg/mL (median (IQR))	452.6 (275.4, 771.9)

Data are presented as mean (standard deviation, SD) or median (interquartile ranges, IQR), depending on data distribution, as well as number, *n* (%).

**Figure 2:**
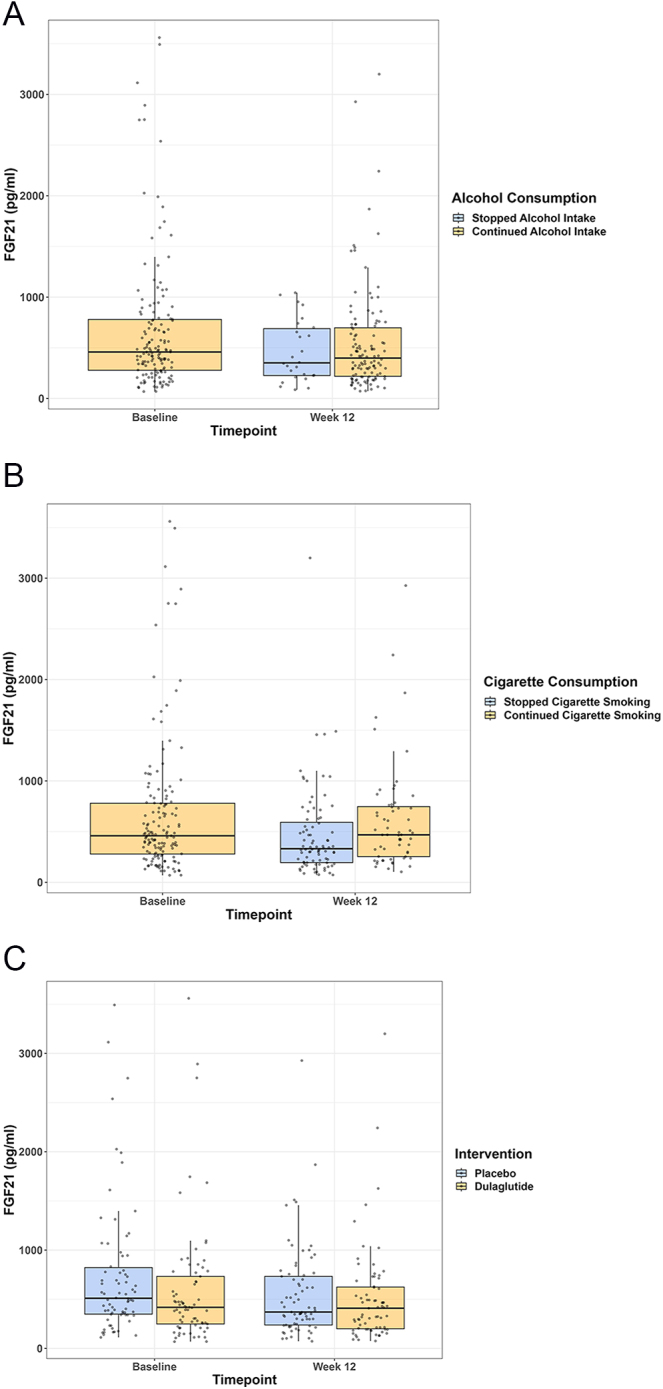
Study 2: FGF21 levels at baseline and week 12 for alcohol consumption status, cigarette smoking status, and intervention. Boxplots represent median (horizontal line) and IQR (25th and 75th percentile, outer box). Whiskers extend to 1.5 times the interquartile range. Single dots represent individual data points.

In participants who stopped drinking alcohol (*n* = 26, 18.1%) during the intervention, plasma FGF21 levels decreased from 565 pg/mL (IQR 450; 822) at baseline to 352 pg/mL (IQR 227; 689) at the 12-week visit. In those who continued drinking (*n* = 118, 81.9%), plasma FGF21 levels decreased from 416 pg/mL (IQR 261; 770) to 399 pg/mL (IQR 219; 698) (estimated difference in FGF21 in alcohol cessation −229 pg/mL (95% confidence interval (95% CI) −440; −15), *P* = 0.03) (see Fig. 2). Adjusting the model for BMI, sex, age, smoking status, and intervention, the results remained statistically significant (see Table 3).

**Table 3:** Study 2: linear mixed-effect model.

Outcome variable FGF21	Estimated difference	95% confidence interval	*P*-value
Alcohol (yes)	229	(15; 440)	**0.03**
Cigarette smoking (yes)	130	(−37; 296)	0.13
Sex (male)	23	(−147; 193)	0.79
Age (years)	14	(8; 20)	**<0.001**
BMI (kg/m^2^)	18	(2; 33)	**0.03**
Intervention (dulaglutide)	−82	(−247; 83)	0.33

Bold indicates statistical significance. Linear mixed-effect model with FGF21 levels as the outcome variable, and alcohol as binary (yes/no), cigarette smoking status (yes/no), sex (male/female), age (years), BMI (kg/m^2^), or intervention (dulaglutide/placebo) as fixed effects, and participants as random effects.

In participants who stopped smoking cigarettes (*n* = 84, 58.3%), plasma FGF21 levels were 439 pg/mL (IQR 266; 737) at baseline and 332 pg/mL (IQR 195; 592) at the 12-week visit, while in those who continued smoking cigarettes (*n* = 60, 41.7%), plasma FGF21 levels were 474 pg/mL (IQR 305; 853) at baseline and 468 pg/mL (IQR 254; 747) at the 12-week visit (estimated difference in FGF21 in smoking cessation 130 (95% CI: −37, 296), *P* = 0.13) (see Fig. 2).

In participants who received the GLP-1 RA dulaglutide, plasma FGF21 levels were 414 pg/mL (IQR 243; 678) at baseline and 409 pg/mL (IQR 200; 624) at the 12-week visit, while in those who received placebo, plasma FGF21 levels were 514 pg/mL (IQR 346; 832) at baseline and 370 pg/mL (IQR 238; 733) at the 12-week visit (estimated difference in FGF21 levels for dulaglutide −82 (95% CI: −247; 83), *P* = 0.33) (see Fig. 2).

FGF21 levels were significantly associated with older age (estimated difference 13.6 (95% CI: 7.8; 19.5), *P* < 0.001) and higher BMI (estimated difference 17.5 (95% CI: 1.7, 33.2), *P* = 0.03) but not with sex (estimated difference 22.9 (95% CI: −146.6; 192.8), *P* = 0.8).

## Discussion

Our analysis has four main findings. First, acute alcohol consumption increases plasma FGF21 levels. Second, alcohol cessation is associated with lower plasma FGF21 levels compared to persistent alcohol consumption. Third, cigarette smoking cessation has no significant impact on plasma FGF21 levels. Fourth, there is no correlation between FGF21 levels and the GLP-1 RA dulaglutide.

In Study 1, we demonstrate a robust, approximately 25-fold increase in plasma FGF21 levels following moderate alcohol consumption in healthy individuals, aligning with previous studies identifying alcohol as a strong inducer of FGF21 secretion (9, 10). The timing and magnitude of the FGF21 response appear to vary across studies, likely attributable to methodical differences in alcohol content and nutritional status, but overall showing a 10- to 40-fold increase ranging from 120 to 250 min with 0.4–0.9 g/kg alcohol (9, 10). Interestingly, we found a delay between peak BAC and peak FGF21. The variation in peak timing across studies suggests a dose-dependent delay in FGF21 secretion relative to the alcohol dose used. In addition, the participants’ nutritional state appears to influence this temporal pattern, with fasted alcohol intake showing an earlier peak in FGF21 levels (10), and concurrent food intake delaying FGF21 increase (23). Mechanistically, the delay may be due to the time required between metabolizing ethanol and alteration in hepatic transcriptional pathways leading to FGF21 gene expression (24). In summary, our findings support the model of FGF21 increase in response to alcohol consumption.

In addition to the acute increase in FGF21 levels in response to alcohol intake, we demonstrate that cessation of social alcohol consumption results in lower FGF21 levels compared to those who continue drinking alcohol. This supports the intriguing possibility raised by preclinical and clinical studies that FGF21 could be used as a marker for liver injury (4). Given the established effects of FGF21 in reducing oxidative stress and liver damage, as indicated by lower AST, ALT, and GGT levels (25), alongside the known increase in FGF21 with alcohol consumption, it can be suggested that once the stressor is removed, metabolism appears to respond by decreasing FGF21, as there is less need to mitigate the harmful effects of alcohol intake. It is known that cessation of alcohol consumption leads to liver tissue recovery (26). We thus speculate that reduced FGF21 levels in individuals who cease drinking alcohol could be an indicator of liver recovery. Further studies investigating FGF21 levels in association with alcohol cessation and liver markers are important to prove this hypothesis.

Contrary to the effects seen after cessation of chronic alcohol intake, we provide evidence that cigarette smoking cessation does not significantly impact FGF21 levels. Previously, cigarette smoking was associated with higher FGF21 levels in humans (14, 15). As of now, the exact mechanism through which smoking leads to elevated FGF21 levels remains unknown. Nakanishi *et al.* suggested that since smoking plays a key role in the pathophysiology of atherosclerosis and metabolic syndrome, both of which are associated with elevated FGF21 levels, it may influence plasma FGF21 levels by contributing to progression of these conditions (14). If smoking itself were the trigger for the FGF21 elevation, one could hypothesize that removing this trigger would lead to a decrease in FGF21 levels. However, our results support Kamizono *et al.*’s findings that cigarette smoking cessation does not lead to a reduction in plasma FGF21 levels (16). One possible explanation for these results may be that, in both studies, smoking cessation within the 12-week study duration was too short to normalize plasma FGF21 levels. Another possible explanation could be that if atherosclerosis and metabolic syndrome, both aggravated by smoking, are the actual triggers for the FGF21 elevation in these subjects, their effects may not be fully reversible or may require more time to subside. Further studies are needed to clarify the association between smoking and FGF21 levels. Finally, it is possible that our sample size was too small, as there is some reduction seen in our studies that does not reach statistical significance.

In addition, we confirm prior findings that FGF21 levels did not differ between GLP-1 RA and placebo (6, 27, 28). Interestingly, we and others identified a correlation of baseline FGF21 and BMI in studies investigating GLP-1 RA (6). These findings suggest that weight loss induced by GLP-1RA may not necessarily be accompanied by a reduction in FGF21. This lack of association may be due to either insufficient metabolic improvement by GLP-1 RAs over the intervention period of 12 weeks to affect FGF21 levels, insufficient changes in key regulatory factors such as hepatic fat, inflammation, and nutritional status, or the presence of FGF21 resistance in obesity and metabolic disease limiting responsiveness to GLP-1 RA.

Furthermore, preclinical data suggest that GLP-1RAs have the potential to reduce both alcohol and nicotine consumption. However, human studies to date show conflicting results. Some studies reported a reduction in alcohol consumption with GLP-1 RAs (29, 30), while others did not (31). Similar conflicting data are described for smoking cessation outcomes, with some studies describing the effect of GLP-1 RAs on smoking cessation (32), while others did not (33). The discrepancy in findings could be due to the use of different GLP-1 RAs, as a class effect has been previously suggested, different participant characteristics that influence effectiveness of GLP-1 RAs, such as type 2 diabetes or overweight, or study settings such as duration of treatment and additional co-medication.

Our analysis has some limitations. In Study 1, sample size was small, and only male participants took part, limiting extrapolation of our findings to acute alcohol intake in women. Furthermore, due to the lack of blood sampling between 240 and 720 min, we cannot draw conclusions regarding FGF21 levels within this timeframe, and we may have missed the peak in FGF21. In addition, the water-intervention was free of glucose. Therefore, fermentable carbohydrates included in beer may have contributed to the observed increase in FGF21 levels in the alcohol-intervention. Nevertheless, our results confirm an increase in FGF21 in response to acute alcohol intake and provide additional evidence about the dynamic changes over time. In Study 2, alcohol consumption was assessed via a self-reported questionnaire, which may not reflect participants’ drinking behavior accurately. In addition, less than 20% stopped drinking alcohol. However, we found lower FGF21 levels, which may be more pronounced in an intervention focused on alcohol cessation. Finally, we only measured total FGF21 and did not measure intact/functional FGF21, which has been done in other studies (10), and did not assess liver markers such as AST, ALT, or GGT, limiting our ability to evaluate liver health.

In conclusion, we provide additional evidence that acute alcohol intake leads to a strong plasma FGF21 increase with a slight delay to blood alcohol peak. In addition, we found that cessation of social alcohol drinking leads to a reduction in FGF21 levels. These findings further contribute to the growing body of evidence suggesting that FGF21 may be a predictor for metabolic health. In addition, we observed no effect of smoking cessation on plasma FGF21 levels. These insights contribute to our understanding of the interplay between alcohol consumption, cigarette smoking, and FGF21 dynamics.

## Declaration of interest

The authors declare that there is no conflict of interest that could be perceived as prejudicing the impartiality of the work reported.

## Funding

Both studies were investigator-initiated. CO Sailer was supported by a grant from the Swiss Academy of Medical Science and by the Goldschmidt & Jacobson Foundation. B Winzeler was supported by a grant from the Swiss National Science Foundation, the Gottfried Julia Bangerter-Rhyner Foundation, the Goldschmidt-Jacobson Foundation, the Hemmi Foundation and by funds of the University of Basel. M Christ-Crain was supported by a grant from the Swiss National Science Foundation (SNF-162608) and the University Hospital Basel, Basel, Switzerland. The funders were not involved in designing, conducting, or analyzing the study, nor in reporting study results.

## Trial registration

ClinicalTrials.gov: Study 1, NCT03883503; Study 2, NCT03204396; Swissmedic Study 2, 2017DR2066.

## References

[1] Brauer M, Roth GA, Aravkin AY, et al. Global burden and strength of evidence for 88 risk factors in 204 countries and 811 subnational locations, 1990–2021: a systematic analysis for the global burden of disease study 2021. Lancet 2024 403 2162–2203. (10.1016/S0140-6736(24)00933-4) 38762324 PMC11120204

[2] Im PK, Wright N, Yang L, et al. Alcohol consumption and risks of more than 200 diseases in Chinese men. Nat Med 2023 29 1476–1486. (10.1038/s41591-023-02383-8) 37291211 PMC10287564

[3] Wang T, Farokhnia M & Leggio L. FGF21 regulates alcohol intake: new hopes on the rise for alcohol use disorder treatment? Cell Rep Med 2022 3 100578. (10.1016/j.xcrm.2022.100578) 35492877 PMC9040172

[4] Spann RA, Morrison CD & den Hartigh LJ. The nuanced metabolic functions of endogenous FGF21 depend on the nature of the stimulus, tissue source, and experimental model. Front Endocrinol 2022 12 802541. (10.3389/fendo.2021.802541) PMC8761941 35046901

[5] Flippo KH & Potthoff MJ. Metabolic messengers: FGF21. Nat Metab 2021 3 309–317. (10.1038/s42255-021-00354-2) 33758421 PMC8620721

[6] Hviid MEB, Christoffersen LAN, Klausen MK, et al. Effect of the GLP-1 receptor agonist exenatide on pro-inflammatory and metabolic biomarkers in individuals with alcohol use disorder: post hoc results from a randomized, double-blinded, placebo-controlled clinical trial. Alcohol Clin Exp Res 2025 49 1659–1666. (10.1111/ACER.70110) PMC12365570 40630018

[7] Zhao C, Liu Y, Xiao J, et al. FGF21 mediates alcohol-induced adipose tissue lipolysis by activation of systemic release of catecholamine in mice. J Lipid Res 2015 56 1481–1491. (10.1194/jlr.M058610) 26092866 PMC4513989

[8] Talukdar S, Owen BM, Song P, et al. FGF21 regulates sweet and alcohol preference. Cell Metab 2016 23 344–349. (10.1016/j.cmet.2015.12.008) 26724861 PMC4749404

[9] Desai BN, Singhal G, Watanabe M, et al. Fibroblast growth factor 21 (FGF21) is robustly induced by ethanol and has a protective role in ethanol associated liver injury. Mol Metabol 2017 6 1395–1406. (10.1016/j.molmet.2017.08.004) PMC5681240 29107287

[10] Søberg S, Andersen ES, Dalgaard NB, et al. FGF21, a liver hormone that inhibits alcohol intake in mice, increases in human circulation after acute alcohol ingestion and sustained binge drinking at Oktoberfest. Mol Metabol 2018 11 96–103. (10.1016/j.molmet.2018.03.010) PMC6001399 29627377

[11] Flippo KH, Trammell SAJ, Gillum MP, et al. FGF21 suppresses alcohol consumption through an amygdalo-striatal circuit. Cell Metab 2022 34 317–328.e6. (10.1016/j.cmet.2021.12.024) 35108517 PMC9093612

[12] Schumann G, Liu C, O’Reilly P, et al. KLB is associated with alcohol drinking, and its gene product β-Klotho is necessary for FGF21 regulation of alcohol preference. Proc Natl Acad Sci U S A 2016 113 14372–14377. (10.1073/pnas.1611243113) 27911795 PMC5167198

[13] Reitsma MB, Fullman N, Ng M, et al. Smoking prevalence and attributable disease burden in 195 countries and territories, 1990–2015: a systematic analysis from the global burden of disease study 2015. Lancet 2017 389 1885–1906. (10.1016/S0140-6736(17)30819-X) 28390697 PMC5439023

[14] Nakanishi K, Nishida M, Harada M, et al. Klotho-related molecules upregulated by smoking habit in apparently healthy men: a cross-sectional study. Sci Rep 2015 5 14230. (10.1038/srep14230) 26382974 PMC4585559

[15] Nakanishi K, Nishida M, Yamamoto R, et al. An implication of Klotho-related molecules in different smoking-related health outcomes between men and women. Clin Chim Acta 2018 476 44–48. (10.1016/j.cca.2017.11.007) 29132901

[16] Kamizono Y, Shiga Y, Suematsu Y, et al. Impact of cigarette smoking cessation on plasma α-klotho levels. Medicine 2018 97 e11947. (10.1097/MD.0000000000011947) 30170389 PMC6392581

[17] Liu D, Pang J, Shao W, et al. Hepatic fibroblast growth factor 21 is involved in mediating functions of liraglutide in mice with dietary challenge. Hepatology 2021 74 2154–2169. (10.1002/HEP.31856) 33851458

[18] Liu J, Yang K, Yang J, et al. Liver-derived fibroblast growth factor 21 mediates effects of glucagon-like peptide-1 in attenuating hepatic glucose output. EBioMedicine 2019 41 73–84. (10.1016/J.EBIOM.2019.02.037) 30827929 PMC6443026

[19] Le TDV, Fathi P, Watters AB, et al. Fibroblast growth factor-21 is required for weight loss induced by the glucagon-like peptide-1 receptor agonist liraglutide in male mice fed high carbohydrate diets. Mol Metabol 2023 72 101718. (10.1016/J.MOLMET.2023.101718) PMC10131131 37030441

[20] Geng L, Lam KSL & Xu A. The therapeutic potential of FGF21 in metabolic diseases: from bench to clinic. Nat Rev Endocrinol 2020 16 654–667. (10.1038/S41574-020-0386-0) 32764725

[21] Chui ZSW, Shen Q & Xu A. Current status and future perspectives of FGF21 analogues in clinical trials. Trends Endocrinol Metab 2024 35 371–384. (10.1016/J.TEM.2024.02.001) 38423900

[22] Kalinowski A & Humphreys K. Governmental standard drink definitions and low-risk alcohol consumption guidelines in 37 countries. Addiction 2016 111 1293–1298. (10.1111/add.13341) 27073140

[23] Desai BN, Singhal G, Watanabe M, et al. Fibroblast growth factor 21 (FGF21) is robustly induced by ethanol and has a protective role in ethanol associated liver injury. Mol Metabol 2017 6 1395–1406. (10.1016/J.MOLMET.2017.08.004) PMC5681240 29107287

[24] Cheong MC, Mackowiak B, Kim HB, et al. Ethanol induction of FGF21 in the liver is dependent on histone acetylation and ligand activation of ChREBP by glycerol-3-phosphate. Proc Natl Acad Sci U S A 2025 122 e2505263122. (10.1073/PNAS.2505263122) 40440069 PMC12146743

[25] Nakanishi K, Ishibashi C, Ide S, et al. Serum FGF21 levels are altered by various factors including lifestyle behaviors in male subjects. Sci Rep 2021 11 22632. (10.1038/s41598-021-02075-8) 34799626 PMC8604971

[26] Thomes PG, Rasineni K, Saraswathi V, et al. Natural recovery by the liver and other organs after chronic alcohol use. Alcohol Res Curr Rev 2021 41 5. (10.35946/arcr.v41.1.05) PMC8041137 33868869

[27] Liu JL & Gao Z. Does GLP-1 suppress hepatocyte glucose production directly, via fibroblast growth factor 21? EBioMedicine 2019 41 5–6. (10.1016/J.EBIOM.2019.03.012) 30876763 PMC6443942

[28] Solomon TPJ, Carter S, Haus JM, et al. Plasma FGF21 concentrations are regulated by glucose independently of insulin and GLP-1 in lean, healthy humans. PeerJ 2022 10 e12755. (10.7717/PEERJ.12755) 35111398 PMC8783558

[29] Probst L, Monnerat S, Vogt DR, et al. Effects of dulaglutide on alcohol consumption during smoking cessation. JCI Insight 2023 8 e170419. (10.1172/JCI.INSIGHT.170419) 37991022 PMC10721313

[30] Hendershot CS, Bremmer MP, Paladino MB, et al. Once-weekly semaglutide in adults with alcohol use disorder: a randomized clinical trial. JAMA Psychiatry 2025 82 395–405. (10.1001/JAMAPSYCHIATRY.2024.4789) 39937469 PMC11822619

[31] Klausen MK, Jensen ME, Møller M, et al. Exenatide once weekly for alcohol use disorder investigated in a randomized, placebo-controlled clinical trial. JCI Insight 2022 7 e159863. (10.1172/JCI.INSIGHT.159863) 36066977 PMC9675448

[32] Yammine L, Green CE, Kosten TR, et al. Exenatide adjunct to nicotine patch facilitates smoking cessation and may reduce post-cessation weight gain: a pilot randomized controlled trial. Nicotine Tob Res 2021 23 1682–1690. (10.1093/NTR/NTAB066) 33831213 PMC8517504

[33] Lengsfeld S, Burkard T, Meienberg A, et al. Effect of dulaglutide in promoting abstinence during smoking cessation: a single-centre, randomized, double-blind, placebo-controlled, parallel group trial. EClinicalMedicine 2023 57 101865. (10.1016/j.eclinm.2023.101865) 36874396 PMC9981899

